# Real time measurement system based on wireless instrumented sphere

**DOI:** 10.1186/2193-1801-2-582

**Published:** 2013-10-31

**Authors:** Yull Heilordt Henao Roa, Fabiano Fruett, Marcos David Ferreira

**Affiliations:** School of Electrical and Computer Engineering, State University of Campinas, Av. Albert Einstein, 400 Campinas, Brazil; Embrapa Instrumentation, São Carlos, SP Brazil

**Keywords:** Acceleration measurements, Impact, Post-harvest, Pseudo-fruit, Virtual instrumentation, Wireless instrumented sphere

## Abstract

In this work, we developed a new measurement system which includes a Wireless Instrumented Sphere (WIS) and a Graphical User Interface (GUI) software, called Real Time Analysis (RTA). This system is able to acquire, process and visualize the three axis acceleration of the WIS allowing the identification and measurements of rotations, vibrations and impacts in real time. The aim of this instrument is to help the fruit producers to reduce food wasting and improve quality, especially in Brazil, one of the major agricultural countries in the world, whose losses could surpass 20% along the post-harvesting handling chain. Additionally, a data Post Processing Analysis software (PPA) provided of a video synchronization option was developed to determine the impact magnitude, position and even the cause of the impact itself (drop, fruit-to-sphere impact, etc.). Both GUIs presented graphics of the three axis acceleration vectors, acceleration magnitude and velocity, as well as the calculations of the number of impacts (peak detection), maximum, minimum and average impact magnitude. The WIS board was encapsulated in the middle of a spherical transparent polyurethane elastomer. It was also intended to be a small, simple, robust and low cost instrument. Its final diameter of approximately 63 mm, 160 g weight and 1.1 relative density. The RTA reduces the time for testing and is suitable for a fast feedback and allows the user to make adjustments in the experiment setup, packing system or even monitor any process along the post-harvesting handling chain, with an immediate response. The PPA with video synchronization option, proved to be a unique tool, relating the acceleration information with the video position.

**Electronic supplementary material:**

The online version of this article (doi:10.1186/2193-1801-2-582) contains supplementary material, which is available to authorized users.

## Introduction

Bruise damage as a result of impact during harvesting, packing, transporting, and handling of fruits has traditionally been identified as a major source of fruit rejection, leading to the loss of profits for the entire fruit industry (Schulte et al. 1992; Marshall and Burgess 1991). Fruit damage can have an immediate low quality consequence in terms of bruise peel wounds and peel compressions, which facilitate the entry of pathogens (*Lasiodiplodia theobromae*, *Penicillium digitatum*), reducing commercial shelf life (García-Ramos et al. 2004a; Fischer et al. 2009).

Impacts commonly occur when the product crosses transfer points among different elements or machines along the commercial packing lines. Bruising occurs when product tissue stress is exceeded. Bruise onset and size depend on a range of factors like height of the transfer points, fruit velocity at impact, hardness of the impact surfaces, curvature of the surfaces, and fruit characteristics such as mass, curvature, temperature, hydration and firmness (García-Ramos et al. 2002).

In order to detect and quantify the impacts suffered by fruits and vegetable during the post-harvesting process, some electronic devices known as pseudo-fruits have already been developed. The Scottish Electronic Potato, developed at the Scottish institute of Agricultural Engineering uses a piezo-electric surface around a molded pseudo-potato (Bollen 2006). The PMS-60 (Pressure Measuring Sphere) developed at the Institute of Agricultural Engineering Bornim in Germany measures the increase of the internal pressure when the instrument is impacted using a pressure transducer (Herold et al. 1996). The Danish Electronic Potato uses a transmitter to relay the signal from a three axis accelerometer to a remote receiver (Canneyt et al. 2003). The Impact Recording Device (IRD) developed in a cooperative research project involving the USDAs Agricultural Research Service and Michigan State University uses a three axis accelerometer as the impact sensor and is presently commercialized by Techmark Inc. USA (Zapp et al. 1990). A wireless instrumented sphere developed at Federal University of Rio Grande do Sul in Brazil, measures compression and impact forces (Muller et al. 2009,2012) and an instrumented sphere developed at the State University of Campinas in Brazil, is able to measure impact, temperature, humidity and position (Nicolau 2009).

Many studies have been carried out to identify critical transfer points in fruit packing lines using pseudo-fruits or Instrumented Spheres (IS), analyzing the characteristics of fruit-to-machine and also fruit-to-fruit impacts. The instrumented spheres help to identify the impact characteristics such as intensity, velocity change and material hardness. Impact data are related to the bruise susceptibility of each fruit by establishing impact damage thresholds of each product (Schulte et al. 1992). The use of IS also helps several authors to suggest ways to improve critical transfer points, including the reduction of fall heights, uses of padding materials, and in some cases the use of decelerators (García-Ramos et al. 2004b).

In this work, we develop a new measurement system including a Wireless Instrumented Sphere (WIS) and a Graphical User Interface (GUI) software, called Real Time Analysis (RTA). This system is able to acquire, process and visualize the three axis acceleration of the WIS allowing the identification and measurements of rotations, vibrations and impacts in real time. The aim of this instrument is to help fruit producers to reduce food wasting and improve quality, especially in Brazil, one of the major agricultural countries and the first world producer of citrus fruit, with 20 million tons/year (FNP 2010), which losses can surpass 20% along the post-harvesting handling chain (Nicolau 2009; Muller et al. 2009).

Additionally a post processing software was developed to make possible the synchronization of the WIS acceleration information and a common video acquisition, providing the exact location of the impacts. The WIS was also intended to be a small, simple, robust and low cost instrument.

## Materials and methods

**Figure 1:**
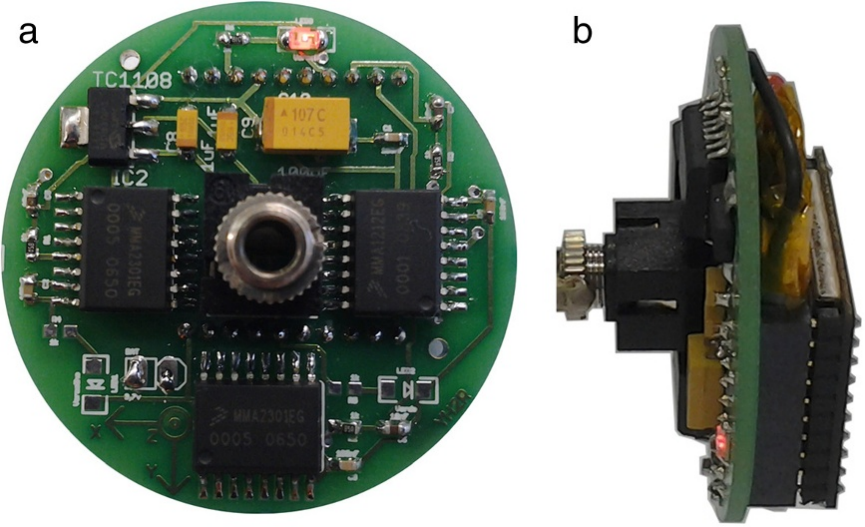
**Wireless Instrumented Sphere board. (a)** Top view. **(b)** Lateral view.

The Real Time Measurement System based on Wireless Instrumented Sphere integrates hardware and software. The hardware is divided into two boards: the first one, called WIS board, includes acceleration sensors, micro-controller, Radio Frequency (RF) module, battery and a connector to charge the battery or to turn off the board. Next, this mobile board is packaged using a spherical molded housing with 63 mm of diameter. The second board, also called base station board, was adapted from a USB-RogerCom board (Messias 2009), and incorporates a radio frequency module. This station board is directly connected to the computer’s USB port, in order to acquire the sensors’ data. Figure 1 shows the WIS board before packaging.

The software is also divided into two parts: the first one, a firmware inside the micro-controller to control the sample rate acquisition, analog-to-digital conversion, data packing and transmission to the station board. The second one is a Graphical User Interface (GUI) developed on a LabVIEW® Virtual Instrument (VI) software, which is a graphical programming language that has been widely adopted throughout industry, academia, and research labs as the standard software for data acquisition and instrument control (Travis and Kring 2006). Two GUI were developed, one for real time operation and the other for post processing operation allowing video synchronization. In the sequence, we detail the WIS hardware and both GUI software.

### Hardware

The WIS electronic circuit uses ratiometric analog Microelectromechanical (MEM’s) acceleration sensors from Freescale™ in a three axis configuration. Two MMA2301 (Freescale 2009b) accelerometers are used for X-axis and Y-axis and one MMA1212D (Freescale 2009a) for Z-axis. These sensors have a full input range of ±200 g and were mounted centrally on a single circuit board within the sphere. We use three single axis accelerometers at the time we developed this prototype because there was not a commercial single three axis accelerometer chip available for this acceleration range. Accelerations are reported in gravity units (g), where 1 g is equivalent to 9.8 m s^–2^. The acquisition, wireless transmission and real time processing of every axis accelerations from ±1 g to ±200 g at a fixed sample rate of 2.2 ms, allow us to identify rotations (±1 g), vibrations (8 g to 10 g) and all kind of impacts (> ± 10 g).

Wireless communication between the WIS and the base station board was achieved with commercial Xbee RF modules from Digi™ (Digi 2006). These modules were introduced in the market in 2006, and were designed to meet the IEEE 802.15.4 standard (ZigBee™). The modules operate within the 2.4 GHz ISM (Industrial, Scientific and Medical) band and transmission range of 50 m.

For a low-cost design, we use the internal micro-controller of the radio frequency module, which includes four analog-to-digital converter channels of 10 bits, internal buffer, and an Application Programming Interface (API), which allows frames transmission to the application containing status packets, as well as source, Received Signal Strength Indicator (RSSI) and payload information from received data packets.

**Figure 2:**
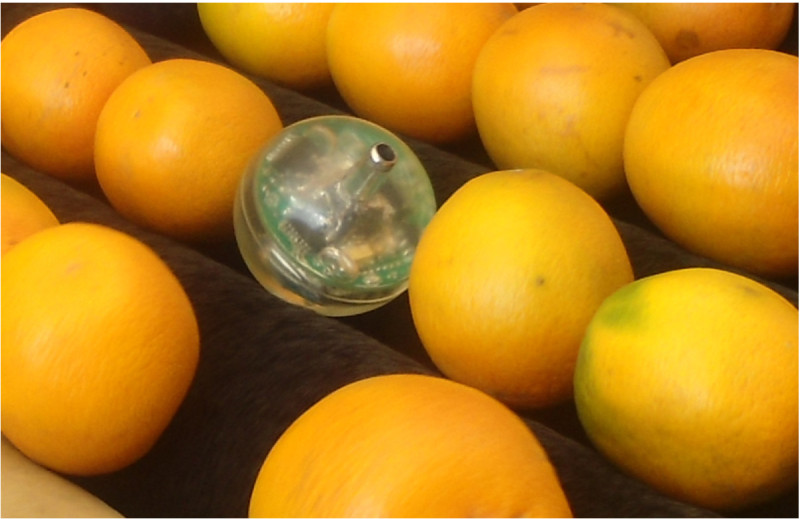
**Wireless Instrumented Sphere (WIS).**

The WIS has only one 3.5 mm connector to charge the 3.7 V lithium prismatic battery. The same connector is also used as a switch to turn off the board when it is not in use. This board was encapsulated in the middle of a spherical transparent polyurethane elastomer (Imuthane with hardness shore A60) and designed to have a final diameter of approximately 63 mm, 160 g weight and 1.1 relative density. Over this 63 mm sphere it is also possible to attach a 3D case with the fruit format, increasing the final diameter near to 70 mm which is considered adequate for experiments with this kind of fruits. Figure 2 represents the Wireless Instrumented Sphere (WIS) among some oranges in a commercial packing line.

### Software

#### Real time analysis (RTA)

**Figure 3:**
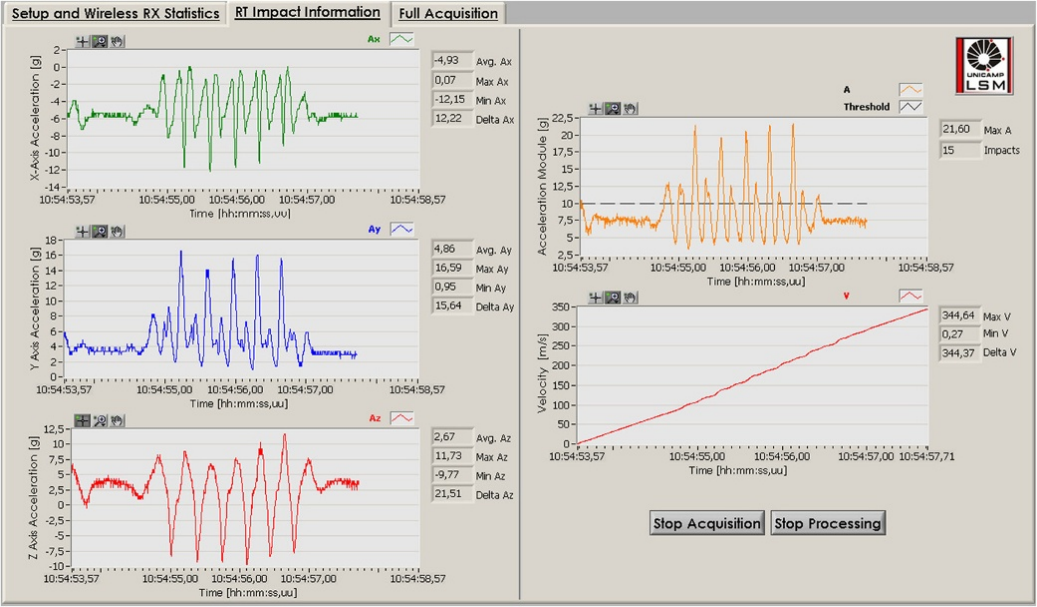
**Real Time Analysis GUI.**

In order to process and analyze all the data sent by the Wireless Instrumented Sphere (WIS) in real time, we developed a software called RTA based on the producer-consumer architecture. This architecture uses two parallel “while loops”, one to receive and temporally store data in the computer RAM memory, and the other to process and save the data. This approach allows the use of a high acquisition sample rate by the WIS. Every frame sent from the WIS to the RTA software is 92 bytes long and contains a start delimiter, data length, API identifier, source address, RSSI, 10 samples of each acceleration axis, and checksum. Figure 3 represents the RTA Graphical User Interface.

The RTA interface is divided into three tabs: *setup and wireless RX statistics*, *RT impact information* and *full acquisition*.

*Setup and wireless RX statistics* allows the user to set the save file path, serial port, time delay for visualization, impact width and impact threshold for peak detection. And also allows the adjustment of the offset and choose the window graph length from 5 to 10 seconds. In the same tab is also presented the wireless communication statistics: RAM buffer, samples per frame, source address, API identifier, total number of frames, Reception (RX) success percentage and Received Signal Strength Indicator (RSSI).

The *RT impact information* tab presents the graphs of the three-axial acceleration vectors, the acceleration magnitude, velocity and the velocity change (total integral of acceleration pulse), as well as the calculations of the number of impacts (peak detection), maximum, minimum and average impact magnitude. All the plots and calculations were made for the window graph length defined in the setup menu. This means that the graphics are plotted in a fixed time window, so when the data fill out the plot, new data will begin to fill it. In the same way, all calculations are only valid for every graph length. In order to get the full visualization and calculations for all the data acquisition, after ending the acquisition process, the user only has to select the *full acquisition* tab.

#### Post processing analysis (PPA)

A post processing software called PPA was additionally developed to process and visualize the impact information in the same way the RTA version does. It also incorporates a video synchronization option which let the user get the precise relationship between the impact acceleration and position of the WIS.

**Figure 4:**
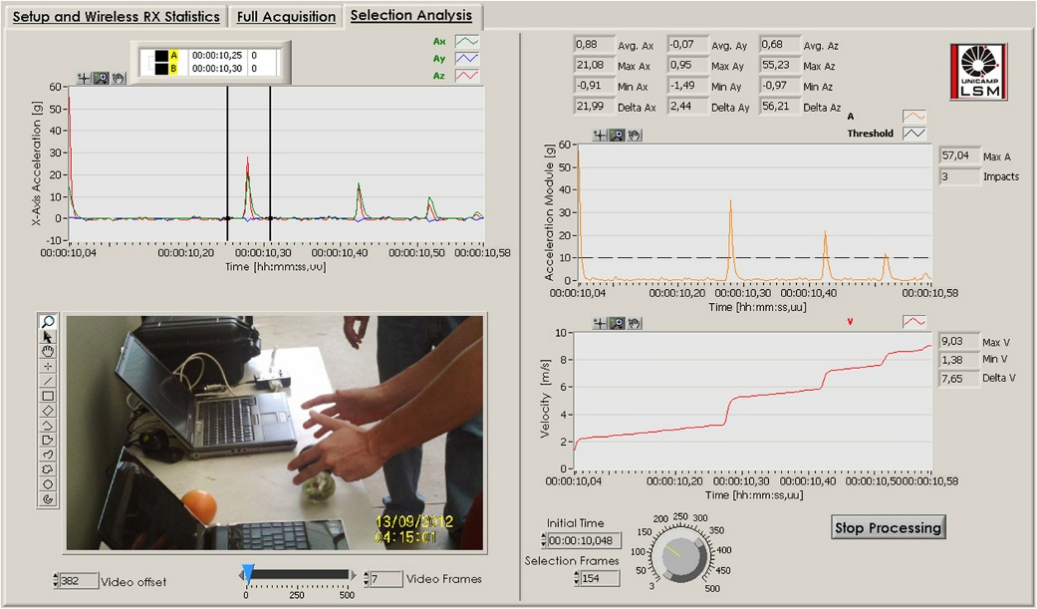
**Post Processing Analysis GUI.**

On this approach, we used a normal 30 Hz video camera and a simple acquisition software to receive and save row data in the computer during the WIS operation. Then, with the PPA software, we synchronized the impact and the video information using an initial drop impact measured by both of them (camera and WIS). This software also allows a manual or automatic video forward or backward frame-by-frame visualization with the corresponding acceleration data. Figure 4 represents the PPA Graphical User Interface.

The *selection analysis* tab of the PPA software, processes the acceleration data of the selected interval, in the same way that the RTA (*RT impact information* tab) does, and it also incorporates the video information and a lean three axial acceleration graph. In this graph, the two parallel cursors indicate the acceleration interval related to the video frame selected.

#### Calibration

In order to overcome the undesirable offset related to the WIS accelerometers, a simple calibration process was developed. This is an interactive process, which takes advantage of the real time processing and the transparent encapsulation material of the sphere. The RT processing allows the measurement of the gravity acceleration in three different positions, aligning every positive acceleration axis (one at a time) to the gravity vector and keeping the other axes almost free of any lateral acceleration. In the RTA the user only has to adjust the offset value of the axis aligned with gravity until the average acceleration reaches 1 g.

#### Tests

The WIS was tested in controlled drop tests and in two orange packing systems. The drop tests were performed to measure the impact peak (acceleration magnitude) for different drop heights and orientations of the WIS. The drop heights were set from the bottom of the sphere to the contact surface and a pneumatic gripper was used to hold the WIS before free-fall release onto a steel plate of 1 cm thickness. Each tests was repeated six times. The orange packing systems were tested using the WIS mixed with oranges (fruit flow), and measured the impact information five times for each packing system.

## Results and discussion

### Impact analysis and drop test

**Figure 5:**
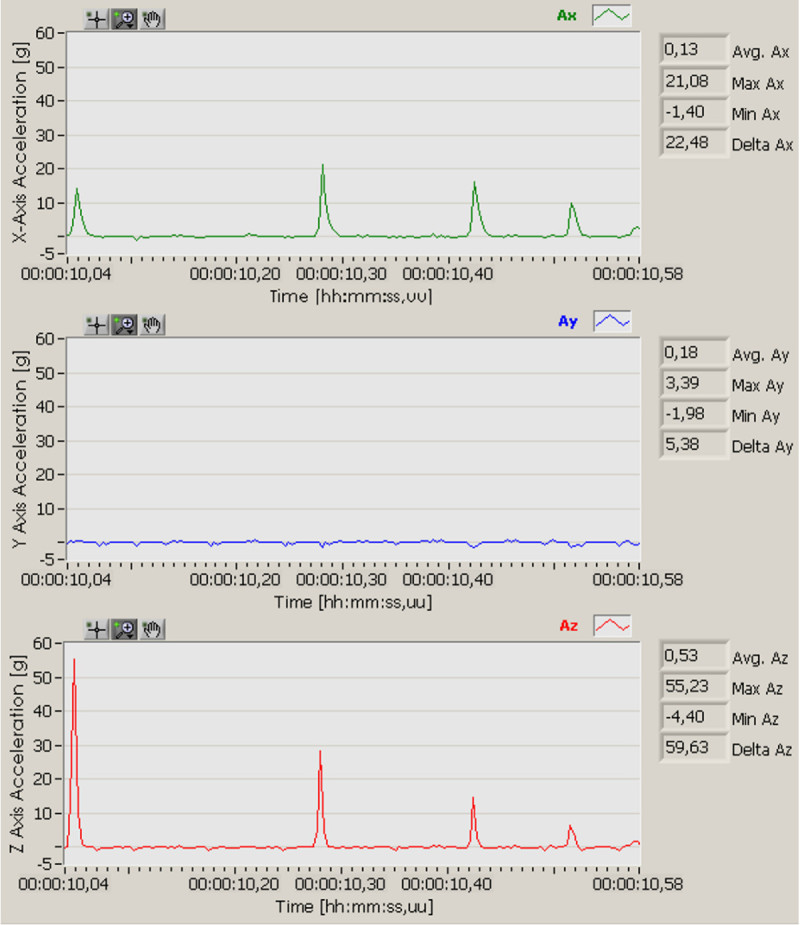
**Acceleration vectors Ax, Ay and Az as a function of time.**

The WIS accelerometer sensors measure the X, Y and Z orthogonal acceleration vectors and the RTA calculates acceleration magnitude and velocity. Figure 5 shows the three acceleration vectors. It also shows four impacts after a drop in X and Z axes, in other words, the initial impact and three impacts generated for the rebound of the instrumented sphere. It is also interesting to note that impacts do not appear in only one axis, even when the sphere was carefully aligned with one of the acceleration axis before the drop.

**Figure 6:**
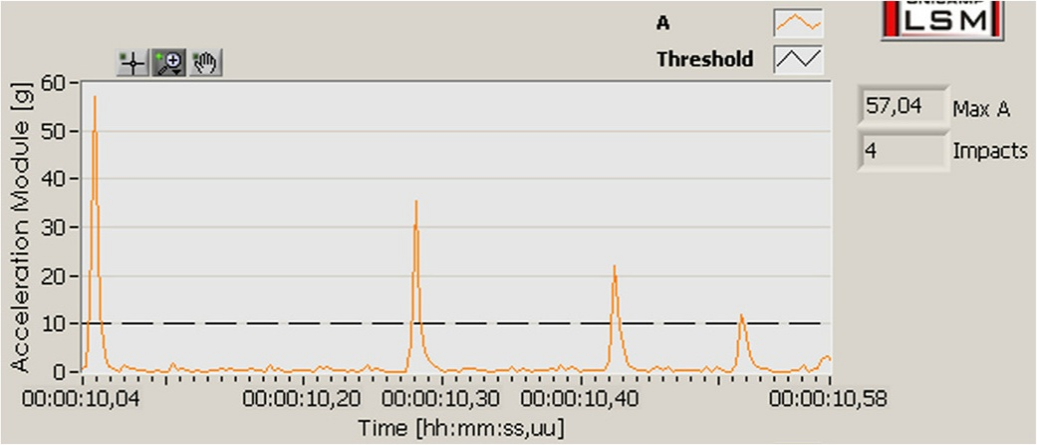
**Acceleration magnitude as a function of time.**

In order to compare the peak acceleration impact, the acceleration magnitude was calculated as shown in Figure 6. In this graph, the four impacts with decreasing magnitude are clearly observed during a second interval. The software also detects the number of impacts below the user-defined threshold. The threshold was fixed at 10 g, which is shown in dashed line.

**Figure 7:**
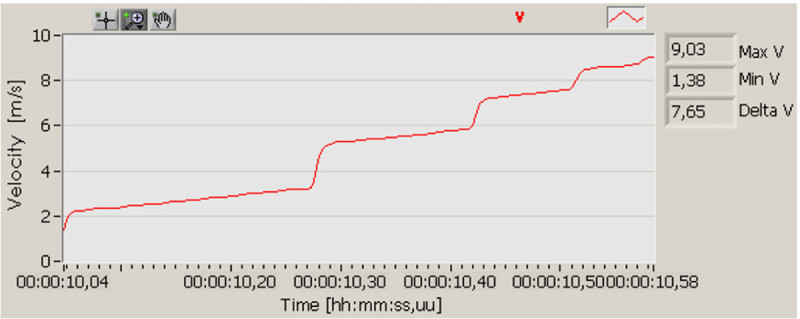
**Velocity change as a function of time.**

Finally, the RTA calculates the total velocity (integral of the acceleration magnitude without initial conditions) in fact, the most interesting part of this graph is the velocity variation occurred under every impact area. It is called velocity change (total integral of acceleration pulse), presented in Figure 7. Peak acceleration and velocity changes are considered the major determining factors on fruit bruising. The importance of these two variables were presented by Zapp et al. (1990) when he claimed that the lower the velocity change for a given peak acceleration impact, the larger the bruise diameter.

**Figure 8:**
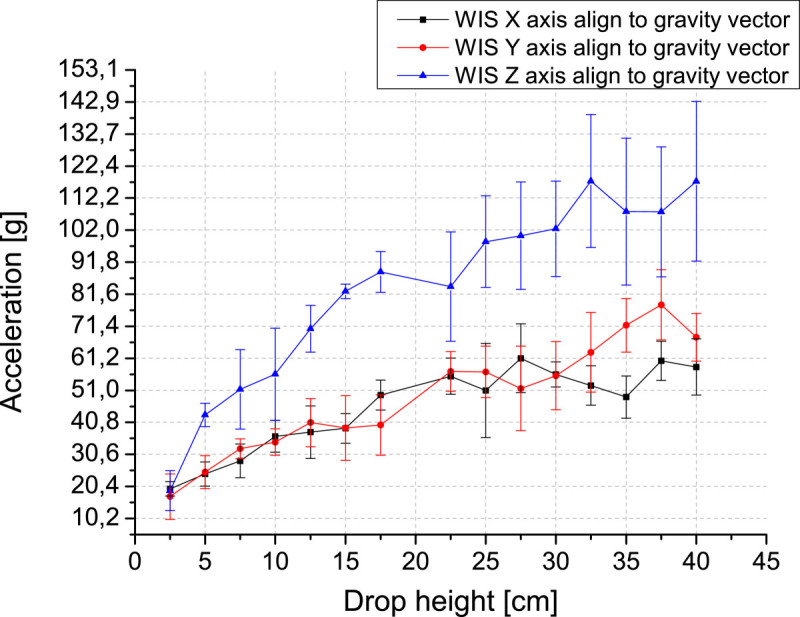
**Acceleration magnitude as a function of drop height.**

We performed six WIS drops aligning one of the acceleration axis to the gravity vector at a time. Figure 8 presents the impact peak (acceleration magnitude) as a function of drop height.

The acceleration appears to be lower in the X and Y axes. We believe this sensitivity difference is related to the thickness variation in of the encapsulation, due to a minor polyurethane width in the center than in the top and bottom of the sphere. This makes the impacts around the center (X-axis and Y-axis) get lower magnitude peaks. If this hypothesis is confirmed, we can design a new Printed Circuit Board (PCB) to obtain a better adjustment of the board into the polyurethane encapsulation process or make a software compensation for the Z-axis data.

### Orange packing system test

**Figure 9:**
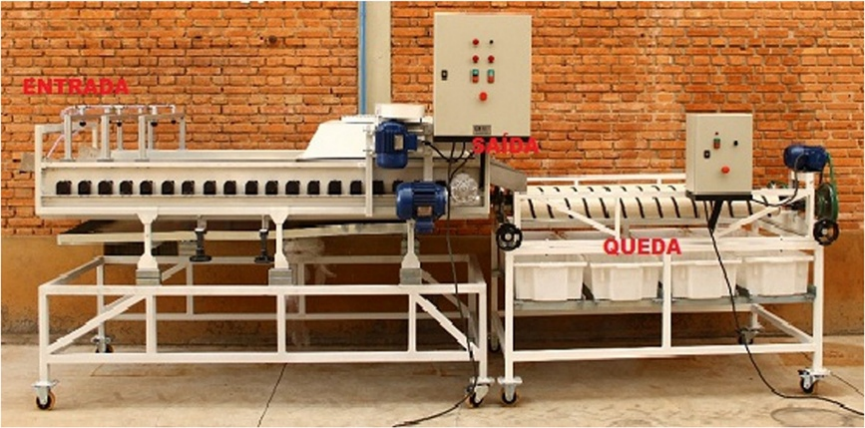
**Horizontal orange packing system.**

The WIS was tested in two orange packing systems. A horizontal and a vertical orange processing and classification system. These tests were made with oranges due to the importance of this fruit in terms of commercial value and international consumption, mainly in São Paulo state that is responsible for 81% of the Brazilian citrus fruit production (Fischer et al. 2009). Figure 9 represents the horizontal packing system.

In both testes, we used the WIS mixed with oranges (fruit flow), and measured the impact information five times for each packing system. According to our expectations, the horizontal packing system presented a reduced number of impacts, very low peak acceleration impacts, and very soft transfer points. To be able to compare the two packing systems we measured the number of impacts above 10 g and 20 g thresholds. This was possible because our sphere transmits all the acceleration data as a function of time in a fixed sample rate. Table 1 presents the impact results for this system.

**Table 1:** **Impact information for horizontal packing system using WIS**

No. of impacts	No. of impacts	Magnitude max.
(>10 g) ($\bar{x}$ ± s.d.)	(>20 g) ($\bar{x}$ ± s.d.)	g ($\bar{x}$ ± s.d.)
9.5 ± 0.57	2.25 ± 1.89	34.48 ± 8.99

The vertical packing system has five critical points. In this case, the WIS was able to identify every critical point, and the impacts produced by the oranges to the sphere normally occurred after a critical point. For this particular system, the video synchronization was very useful to identify the real cause of the impact. Table 2 presents the results for critical points using WIS. We choose not to show the vertical packing system figure, due an ongoing patenting process.

**Table 2:** **Critical points in vertical packing system**

Transfer points	Magnitude
	g ($\bar{x}$ ± s.d.)
Entrance	12.36 ± 4.01
Drop 1	33.29 ± 5.02
Drop 2	20.58 ± 5.38
Drop 3	46.32 ± 10.01
Output	22.74 ± 2.62

In Table 3, we also presented the impact information summary. The vertical packing system presents more number of impacts above 10 g and 20 g thresholds and a higher peak acceleration impact than the horizontal packing system.

**Table 3:** **Impact information for vertical packing system using WIS**

No. of impacts	No. of impacts	Magnitude max.
(>10 g) ($\bar{x}$ ± s.d.)	(>20 g) ($\bar{x}$ ± s.d.)	g ($\bar{x}$ ± s.d.)
26 ± 4.96	14.5 ± 4.7	56.67 ± 8.02

## Conclusions

The real time wireless instrumented sphere measurement system, proved to be a very valuable tool for impact analysis. This development has resulted in a complete system with a small, simple, robust and low cost WIS which includes two software, one for real time analysis and the other for post processing analysis provided with a video synchronization option. Both GUI presented graphics of the three axial acceleration vectors, acceleration magnitude, velocity and the velocity change, as well as the calculations of the number of impacts, maximum, minimum and average impact magnitude for all the graphs.

The Real Time Analysis software (RTA) allows the user to visualize all the graphs and calculations at the same time the sphere is measuring acceleration data. This new approach reduces the time for testing and is suitable for a fast feedback, allowing the user to make adjustments in the experiment setup, packing system or even monitor any process along the post-harvesting handling chain, with an immediate response.

The Post Processing Analysis software (PPA) with video synchronization option, proved to be a unique tool, relating the acceleration information with the video position. This provides the user a clear idea of the impact magnitude, position and even the cause of the impact itself (drop, fruit-to-sphere impact, etc.). It is also a very useful tool to visualize and remember the tests conditions, especially if the data are not immediately processed.

The WIS continues acquisition and wireless transmission, in addition to the RTA real time processing of every axis acceleration data as a function of time at a fixed sample rate. It allows us to identify rotations (±1 g), vibrations (8 g to 10 g) and all kind of impacts (> ± 10 g).

## Authors’ original submitted files for images

Below are the links to the authors’ original submitted files for images. Authors’ original file for figure 1 Authors’ original file for figure 2 Authors’ original file for figure 3 Authors’ original file for figure 4 Authors’ original file for figure 5 Authors’ original file for figure 6 Authors’ original file for figure 7 Authors’ original file for figure 8 Authors’ original file for figure 9 Authors’ original file for figure 10
