# Supplementary figures and images for: Real time measurement system based on wireless instrumented sphere
Source: Springerplus. 2013 Oct 31;2(1):582. doi: 10.1186/2193-1801-2-582 (PMC4320251; doi:10.1186/2193-1801-2-582)

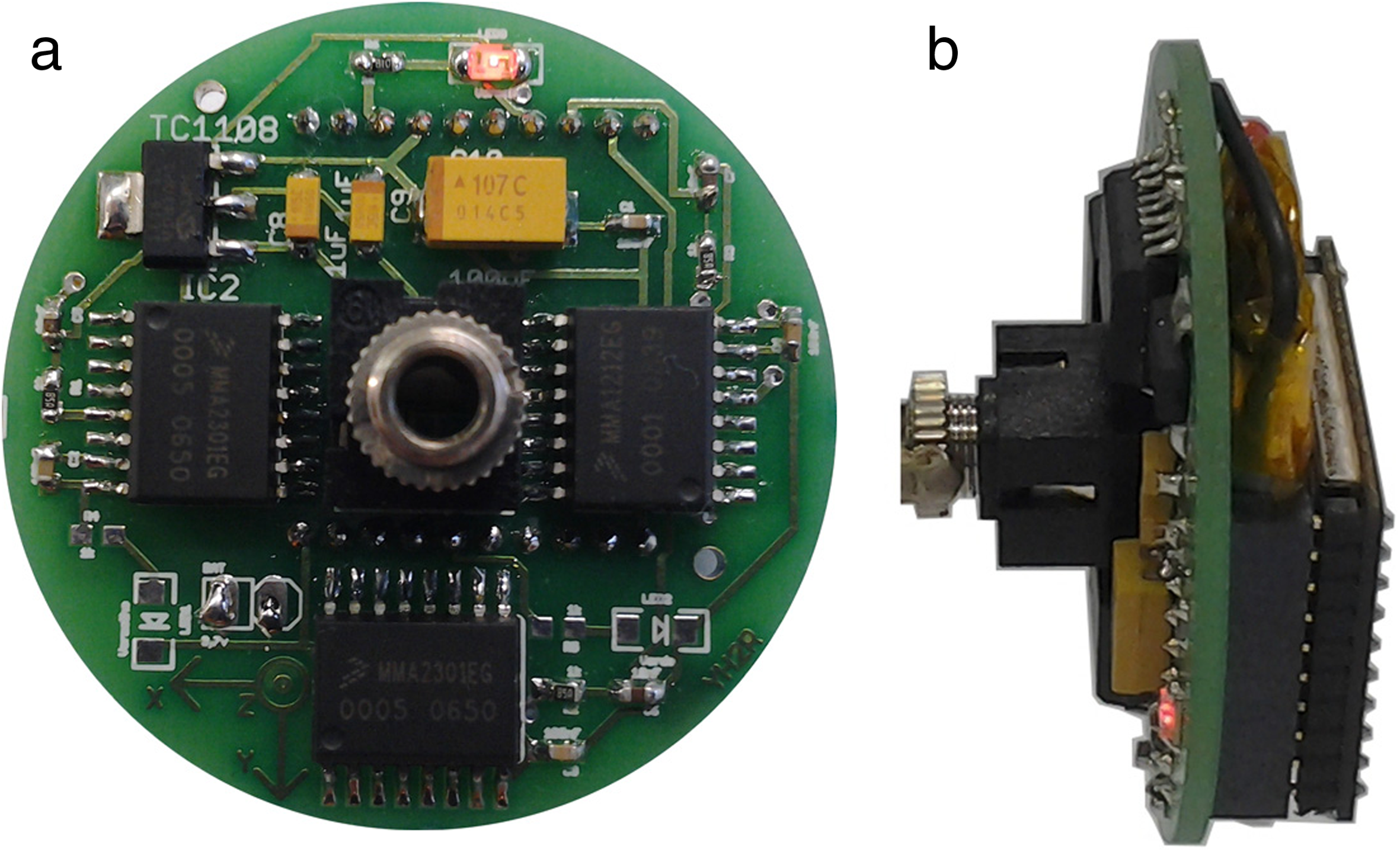

Supplement: Supplementary file 1 — Authors’ original file for figure 1 [file 40064_2013_1435_MOESM1_ESM.tiff]

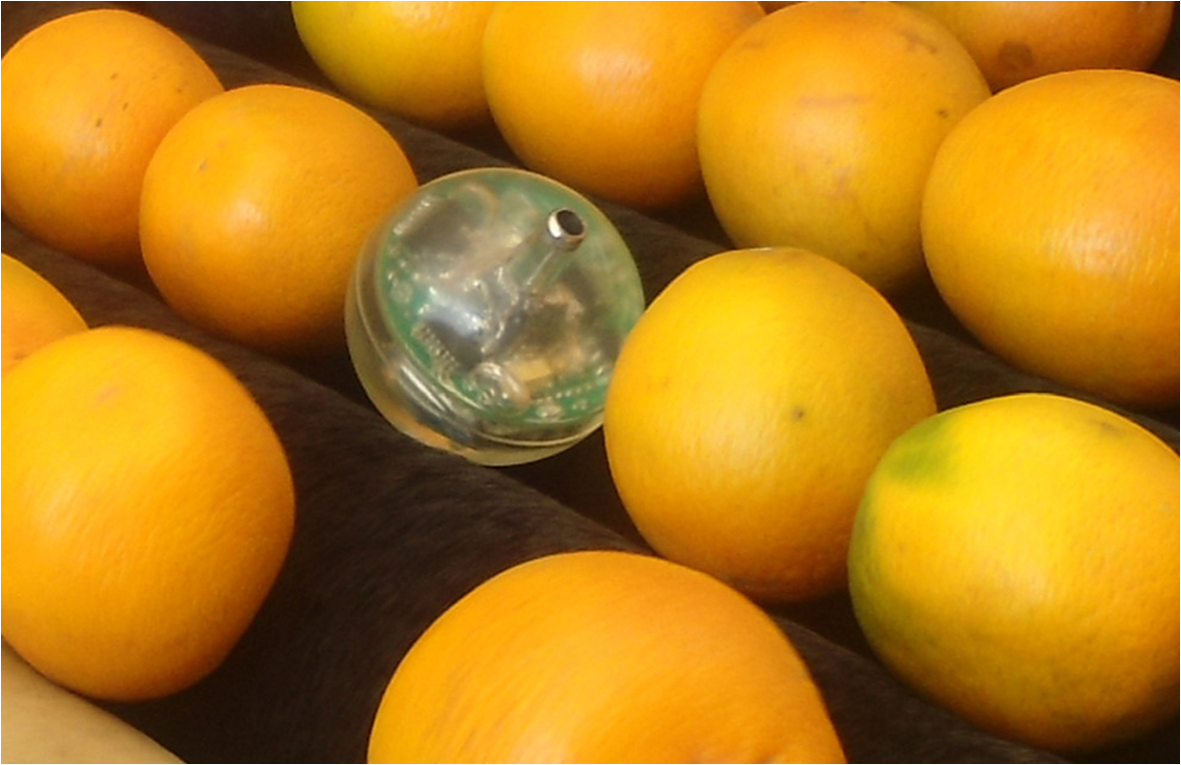

Supplement: Supplementary file 2 — Authors’ original file for figure 2 [file 40064_2013_1435_MOESM2_ESM.tiff]

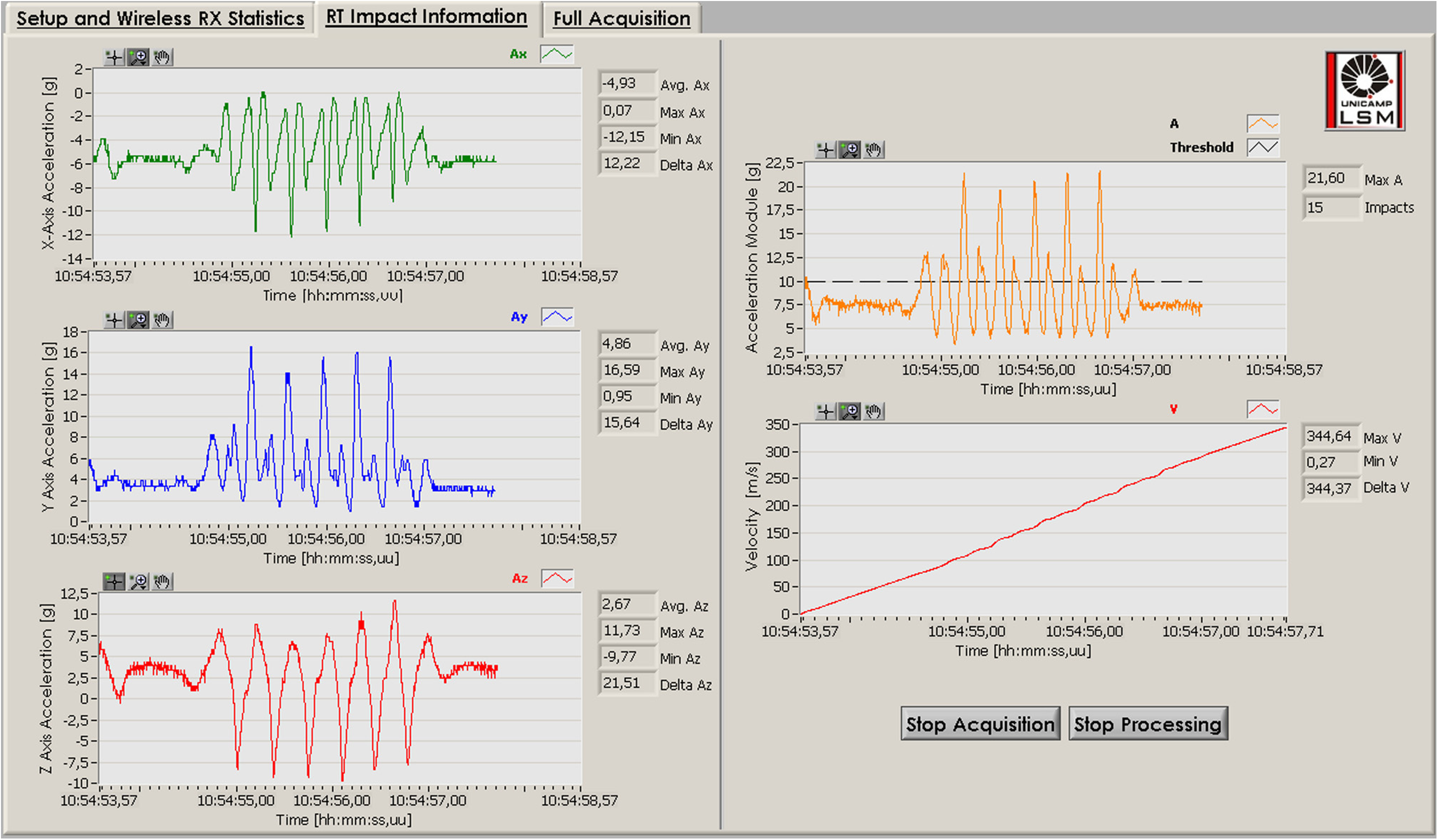

Supplement: Supplementary file 3 — Authors’ original file for figure 3 [file 40064_2013_1435_MOESM3_ESM.tiff]

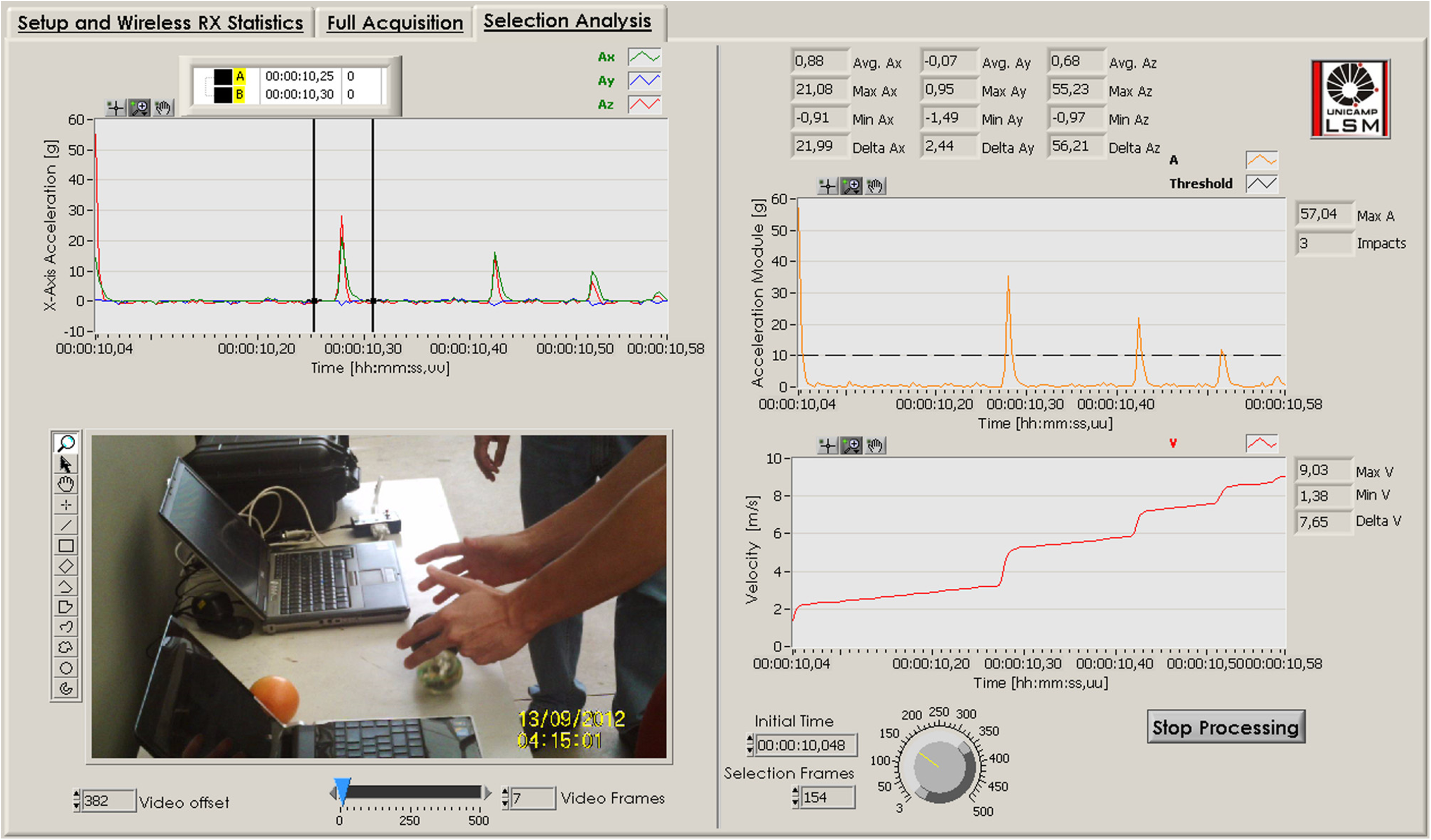

Supplement: Supplementary file 4 — Authors’ original file for figure 4 [file 40064_2013_1435_MOESM4_ESM.tiff]

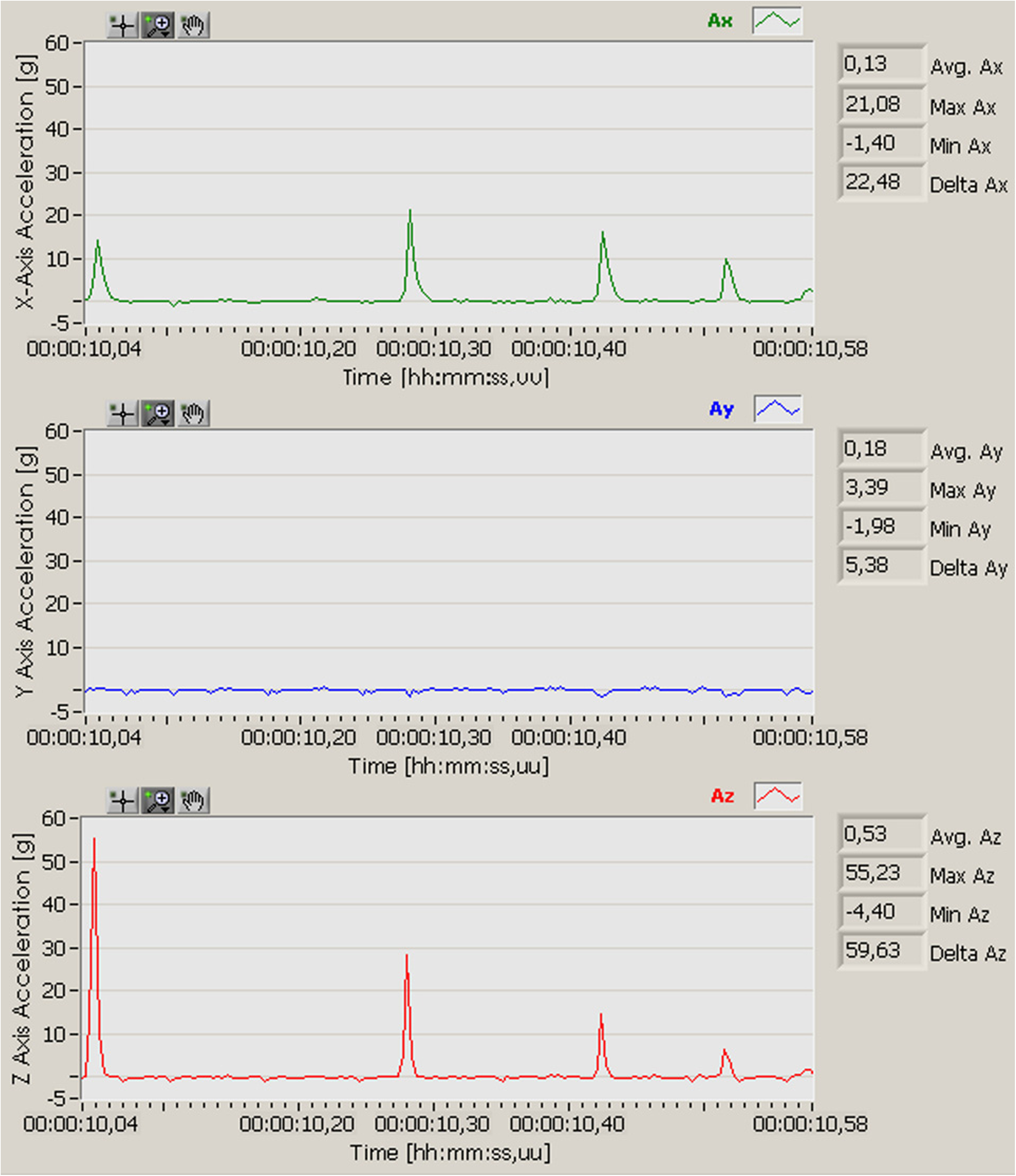

Supplement: Supplementary file 5 — Authors’ original file for figure 5 [file 40064_2013_1435_MOESM5_ESM.tiff]

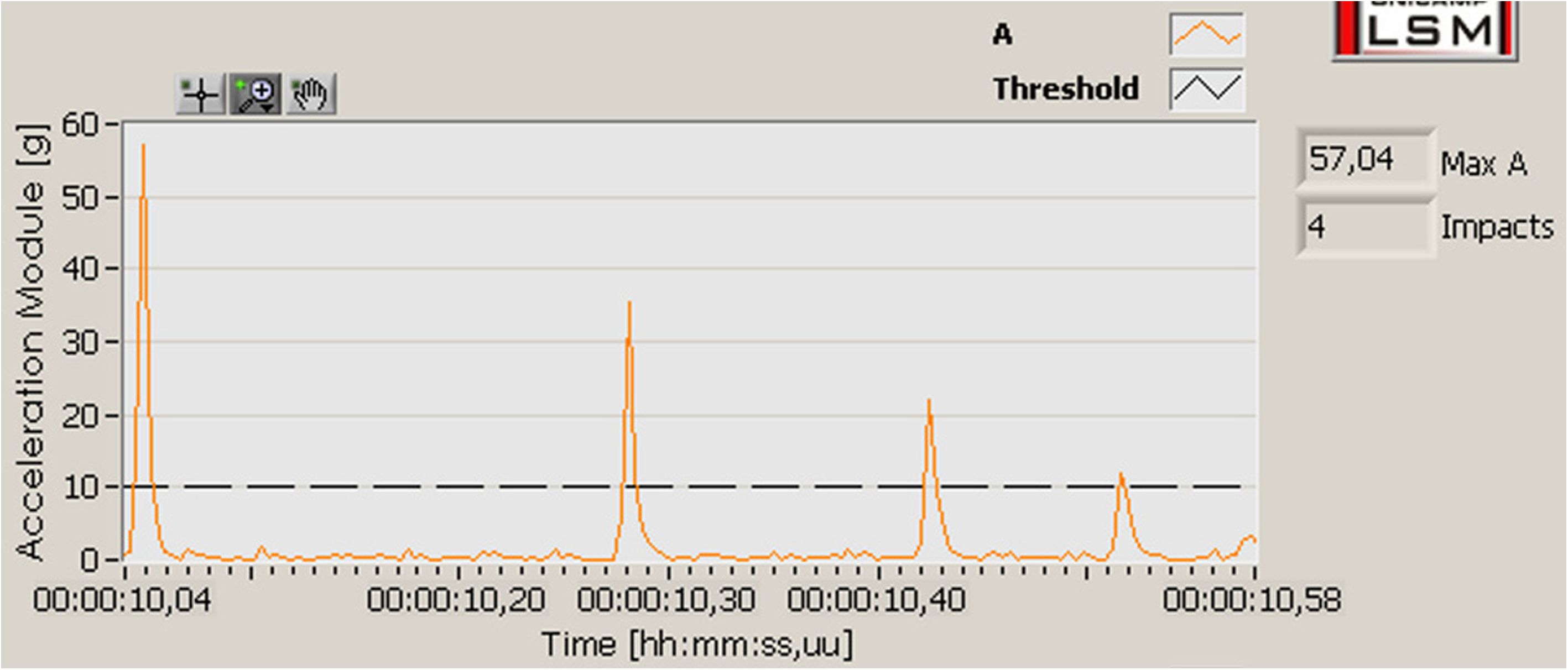

Supplement: Supplementary file 6 — Authors’ original file for figure 6 [file 40064_2013_1435_MOESM6_ESM.tiff]

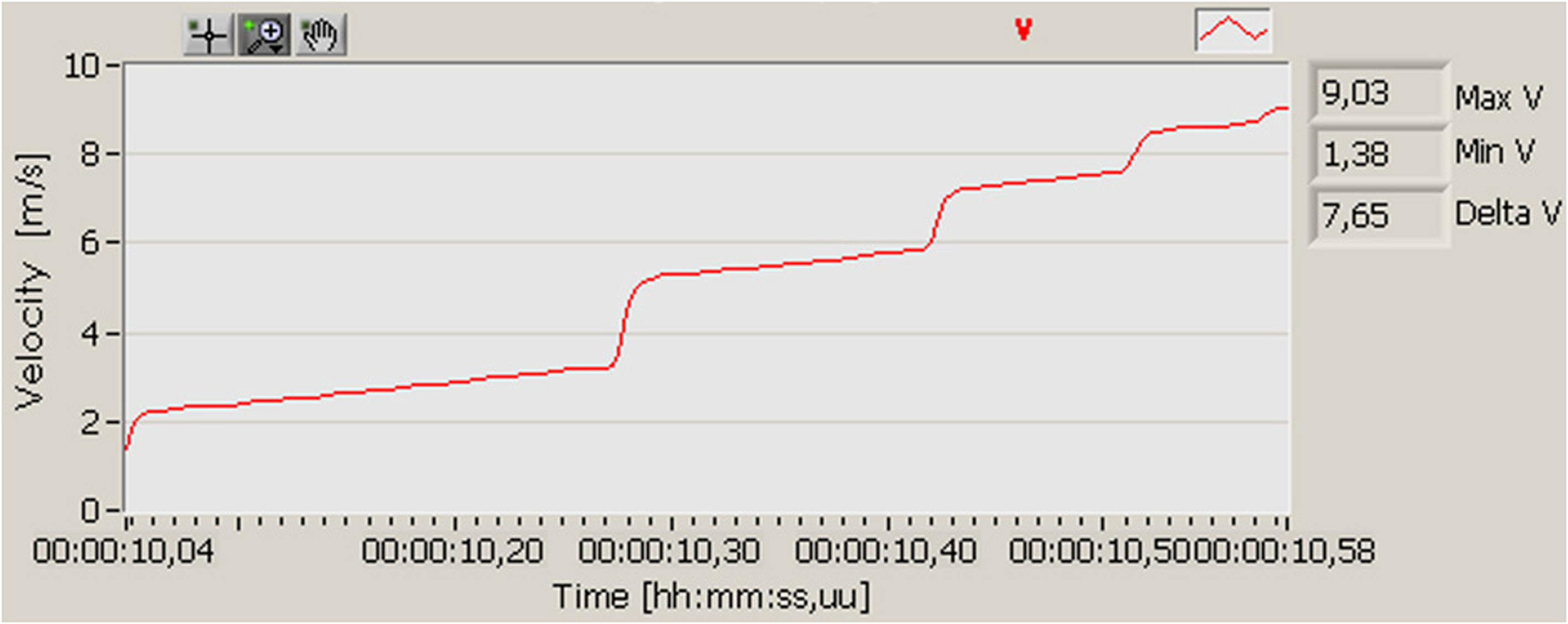

Supplement: Supplementary file 7 — Authors’ original file for figure 7 [file 40064_2013_1435_MOESM7_ESM.tiff]

Acceleration [g]

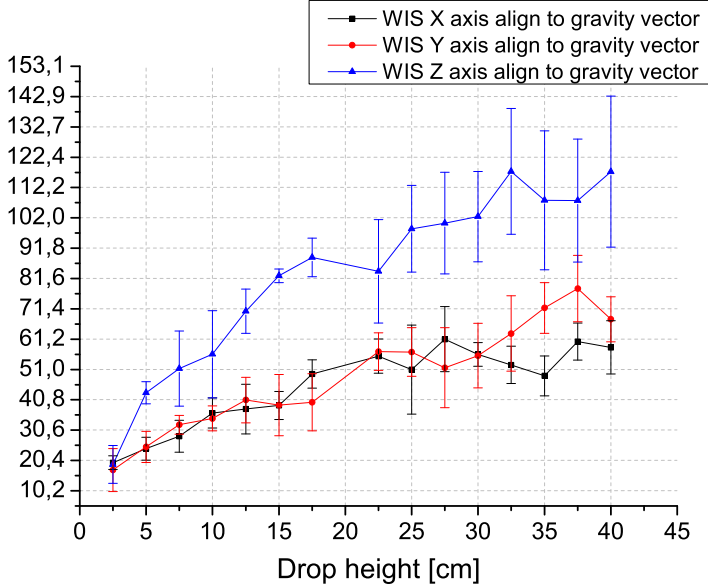

Supplement: Supplementary file 8 — Authors’ original file for figure 8 [file 40064_2013_1435_MOESM8_ESM.pdf]

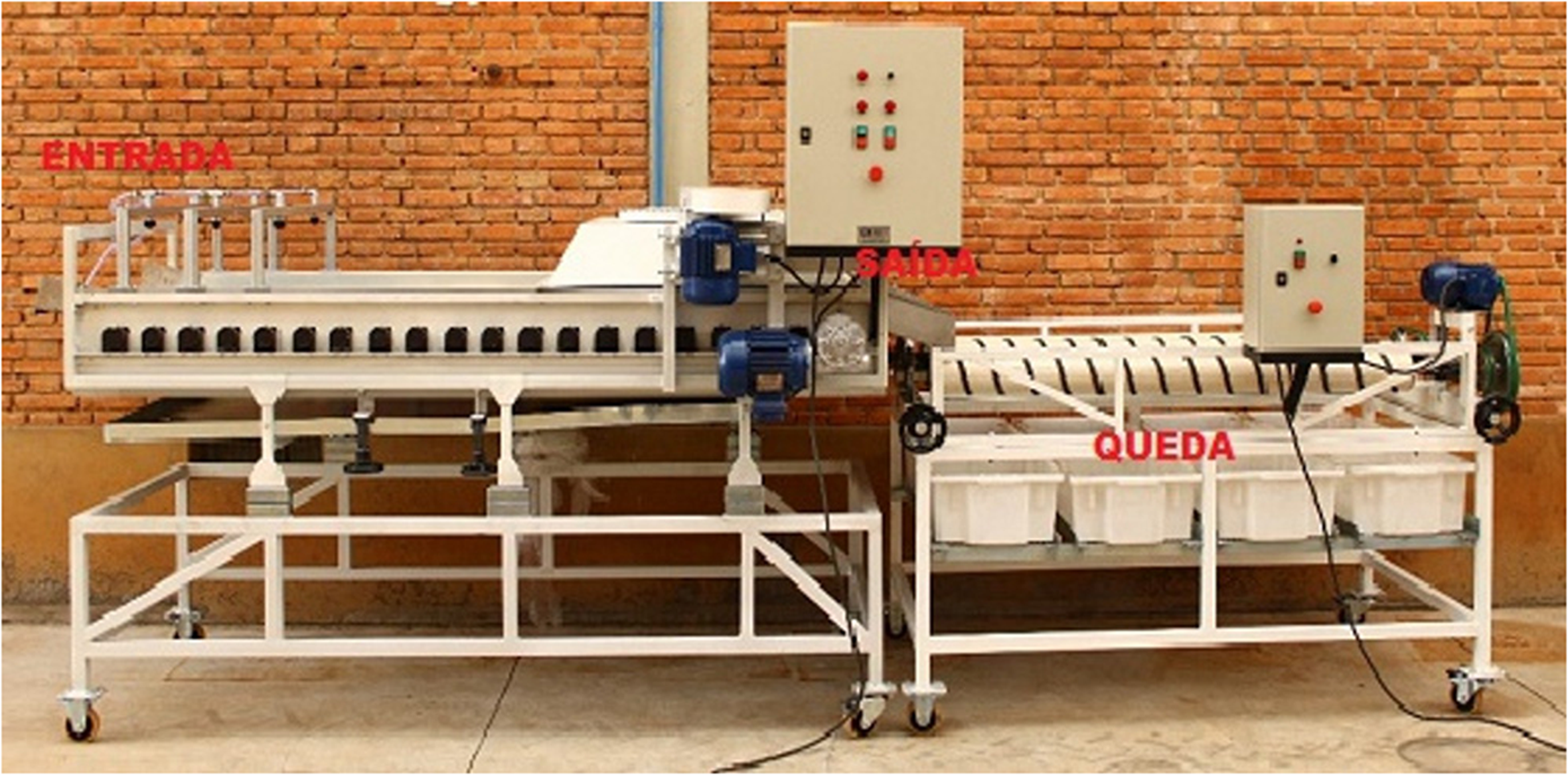

Supplement: Supplementary file 9 — Authors’ original file for figure 9 [file 40064_2013_1435_MOESM9_ESM.tiff]
